# MRI Brain Tumor Image Classification Using a Combined Feature and Image-Based Classifier

**DOI:** 10.3389/fpsyg.2022.848784

**Published:** 2022-03-04

**Authors:** A. Veeramuthu, S. Meenakshi, G. Mathivanan, Ketan Kotecha, Jatinderkumar R. Saini, V. Vijayakumar, V. Subramaniyaswamy

**Affiliations:** ^1^Department of Information Technology, School of Computing, Sathyabama Institute of Science and Technology, Chennai, India; ^2^Department of Information Technology, Jeppiaar SRR Engineering College, Chennai, India; ^3^Department of Information Technology, School of Computing, Sathyabama Institute of Science and Technology, Chennai, India; ^4^Symbiosis Centre for Applied Artificial Intelligence, Symbiosis International (Deemed University), Pune, India; ^5^Symbiosis Institute of Computer Studies and Research, Symbiosis International (Deemed University), Pune, India; ^6^School of Computer Science and Engineering, University of New South Wales, Sydney, NSW, Australia; ^7^School of Computing, Shanmugha Arts, Science, Technology & Research Academy Deemed University, Thanjavur, India

**Keywords:** brain tumor, classification, deep neural network, actual image, segmented image, combined feature and image based classifier (CFIC)

## Abstract

Brain tumor classification plays a niche role in medical prognosis and effective treatment process. We have proposed a combined feature and image-based classifier (CFIC) for brain tumor image classification in this study. Carious deep neural network and deep convolutional neural networks (DCNN)-based architectures are proposed for image classification, namely, actual image feature-based classifier (AIFC), segmented image feature-based classifier (SIFC), actual and segmented image feature-based classifier (ASIFC), actual image-based classifier (AIC), segmented image-based classifier (*SIC*), actual and segmented image-based classifier (ASIC), and finally, CFIC. The Kaggle Brain Tumor Detection 2020 dataset has been used to train and test the proposed classifiers. Among the various classifiers proposed, the CFIC performs better than all other proposed methods. The proposed CFIC method gives significantly better results in terms of sensitivity, specificity, and accuracy with 98.86, 97.14, and 98.97%, respectively, compared with the existing classification methods.

## Introduction

The general health of the people and livestock and nature, in general, is considered the foremost wealth component of any nation. Thus, improving health and controlling diseases are crucial factors in the sustenance and progress of the world. Early identification of diseases is crucial in disease control. Therefore, the rapid and accurate diagnosis is of foremost significance. There has been a steady growth in the medical instrumentation field in the past and present centuries. With the advent of computers, accurate interpretation of data analysis and measurements has led to vast improvement. Computer-aided analytical tools have become a great help to medical experts in decision-making. Computer-aided diagnosis is a fast-growing research area. Medical image processing (MIP) is one of the important techniques in diagnosis, where classification is a very important process to classify the disease, whether benign or malignant.

Successful treatment of diseases depends largely on early detection and accurate assessment of the state of the disease. Disease diagnosis is an important process in the maintenance of global well-being. Modern diagnosis involves measurement, analysis, and decision-making. Computers play a critical role in medical decision support. Among the fatal diseases, brain cancer is particularly difficult because it is not usually detected until it is too late for prognosis. MRI is focused very specifically because of its effectiveness and harmlessness. The brain tumor classification is essential for the treatment plan and further the assessment process of the tumor.

In this study, we have proposed and investigated seven different classifiers to efficiently classify the MRI brain tumor images into benign or malignant types. The deep learning (DL)-based neural network architecture is designed to group each pixel into a voxel of interest, capturing the required information by choosing the input to be applied in the deep network. The choice of input contributes to avoiding overfitting and reducing computational complexity significantly. Selecting the local precision and global spatial intensity features are considered as most important in this study. The experimental results are validated using the evaluation metrics such as sensitivity, specificity, and accuracy. Among the seven classifiers, the combined feature and image-based classifier (CFIC) performs significantly better than the existing classification methods.

The remaining sections are organized as follows: related works using various methods are discussed in image classification, proposed work of the various classifiers are presented, results and discussions of the proposed methods are compared, and finally, the conclusion and future works are discussed.

## Related Works

Various methods are available for brain tumor classification. Among various methods, neural network-based methods, convolutional neural network (CNN) methods, and DL methods are widely used.

The study has investigated the use of the deep features extracted from the CNNs that are pretrained in the prediction of survival time. It provides further evidence for domain-specific fine-tuning to improve its performance. Standard dataset is available in the internet. It has an accuracy of approximately 81% in the case of leave-one-out-based cross-validation (Ahmed et al., 2017).

The study has proposed a hybrid method for classifying the tissues of the brain tumor image. In this technique, the system employs a genetic algorithm (GA) for feature extraction and an support vector machine (SVM) for classification purposes. The features are further compared with the stored features, and the method is used to capture the images and their visual contents. This method signifies the raw image to simplify decision-making in a very focused form. The choice of the features composed is very difficult in the classification methods that are duly solved using the GA. The features with the SVM have been used to classify the tumor as normal or abnormal. If the tumor is detected based on mean, mode, and median, then the tumor is classified as either meningioma or pituitary tumor. The performance of the algorithm is assessed based on the images containing the brain tumor (Bangare et al., 2017).

The study has found the solutions for classifying MRI brain tumors using the hybrid technique. This method gives a second opinion for the physicians to succeed in the treatment process. It works only on the specific type of tumor with limited image datasets (Mohan and Subashini, 2018).

The study has proposed a simple three-step algorithm for identifying brain tumors in MRI images. The process consists of identifying patients with brain tumor, automatically selecting all the abnormal slices of such patients, thereby segmenting, and recognizing the tumor. The features are extracted using the discrete wavelet transform (DWT) on several normalized images, which are further classified using the SVM algorithm for the first process. The same procedure is followed for the second process. The random forest (RF) classification algorithm is used instead of SVM. Another 400 subjects are divided into a ratio of 3:1 without any overlap. All the T2-weighted slices were configured as one single image-based classification. It was equipped with a new and unique contralateral approach, with patch thresholds for the segmentation process. This patch threshold does not require training sets or templates used for the investigations of segmentation. The tumors are segmented with high accuracy compared with the other classification methods (Gupta et al., 2017).

The study has found that deep learning is a new approach in the machine learning field, which is applied in many complex application areas. This DL approach, combined with DWT and principal component analysis (PCA), leads to better brain tumor classification performance. The lack of a real-time dataset for getting better results is less and less convenient in time (Mohsen et al., 2018).

The study has presented the deep feature and machine learning-based MRI brain tumor image classification method to classify the MRI brain tumor images into benign and malignant. The different CNN classifier models are compared with the deep feature and machine learning classifier models, and better result is achieved (Kang et al., 2021). The local constraint-based convolutional dictionary learning is the new method to classify brain tumors into normal or abnormal types. This supervised classification method uses the REMBRANDT datasets for classification purposes. This method helps to classify the tumor significantly better than the various existing methods (Gu et al., 2021).

The study has presented the modified deep convolutional neural network (DCNN) to efficiently classify brain tumor images into the benign and malignant. The MRI brain images of T1, T2, and T2 fLuid-attenuated inversion recovery (FLAIR) of 220 cases are used to classify using this method with a better accuracy rate (Hemanth et al., 2019). The study has discussed the DL-based opposition crow search (DL-OCS) method to efficiently classify MRI brain tumor images. In this technique, the optimal feature selection is considered as most important for the classification process. The DL-OCS method performs better in terms of sensitivity (86.45%), specificity (100%), and accuracy (95.22%) (Raj et al., 2020).

The study has discovered various key research fields in medical image analysis using machine learning algorithms. The machine learning algorithms are helped to maintain the medical records in digital formats, increasing over time. The advantages of digitizing the medical images are it is accurate, up-to-date, quickly accessible, more reliable, easy to share, enhances privacy and security, reduces costs, etc. The important issue is the lack of datasets for training in medical image analysis. Furthermore, rare diseases are difficult to be spotted with reliable accuracy due to data imbalance or lack of training samples (Ker et al., 2018).

The study has presented the new progressive growing generative adversarial network (PGGAN) method based on CNN, which is an augmentation method that allows the maximization of the learning optimization of the network. The performance of the PGGAN method in terms of accuracy has been increased considerably (Khan et al., 2020). The study has presented a CNN with image processing and data augmentation methods to classify the MRI brain tumor images into cancerous or non-cancerous. The experiments have been carried out with very small datasets, compared with various existing transfer learning methods, which show better performance (Ahmed et al., 2021). The study has presented the new CNN approach for T1-weighted MRI brain tumor image classification. Then network was tested using the 10-fold validation techniques. It gives better accuracy than the traditional classification methods (Badza and Barjaktarovic, 2020).

The study has discussed two different classifiers based on the neural networks methods for MRI brain tumor images. This method consists of three stages: feature extraction, selection, and classification. The features are extracted using DWT, and the most relevant features are selected using PCA methods. The classification process is the final stage based on supervised learning techniques. They are feed-forward artificial neural networks and backpropagation neural network-based methods, and all these methods help classify the tumor into normal or abnormal MRI based on the brain tumor images (Kharat et al., 2012).

The study has proposed a method for segmenting and classifying the MRI brain tumor. The input MRI image is preprocessed by using the Ostu method for choosing the threshold. Furthermore, the tumor is detected using the K-means clustering. The image features are extracted using DWT and the Gabor wavelet. The PCA was used to reduce the feature set. Finally, the SVM classifier is used to classify the tumor image as benign or malignant (Mathew et al., 2017).

The study has found that many neurological diseases are identified using MRI images rather than other images. Early detection of brain tumors saves human life. Deep neural network (DNN)-based algorithms are widely used for various applications, especially for brain tumor segmentation and classification tasks. In this study, the deep wavelet autoencoder combined with DNN helps to reduce the feature dataset and gives better performance (Mallick et al., 2019).

Classification is a crucial task in data mining and has many applications. There are several applications in computer vision for this challenging task. Data belonging to different types can be classified using this approach to categorize every item within a dataset into a group or class that is predetermined. One set of the training records is used for the problem definition so that every record is named with a class value extracted from a set of k varied discrete values indexed by {1,., k}. These training data are utilized to formulate the classification models relating to features in an underlying record to one class of labels. For example, this training model can be utilized for forecasting the record to a class label when the class of an instance is unknown (Sravani et al., 2014).

## Proposed Works

The MRI brain tumor image classification is a process that plays a vital role in identifying and classifying dangerous diseases, either benign or malignant. Research has been conducted to diagnose brain tumours based on medical images. Two complementary qualities are required for strong classification performance, which are the descriptiveness and discriminativeness of the extracted features. In classification, machine learning is critical because of its wide range of approaches and suitability to a given problem.

### Deep Neural Network Architecture

The DNN used in this study is a simple extension of classical artificial neural network (ANN) with many hidden layers. The input layer receives the selected feature vectors of each image from the training dataset. It is augmented with the bias neuron, which adds a constant bias signal of +1 to each feature vector. Each neuron in the input layer merely distributes its feature signal to all the first hidden layer nodes through a weight.

**FIGURE 1:**
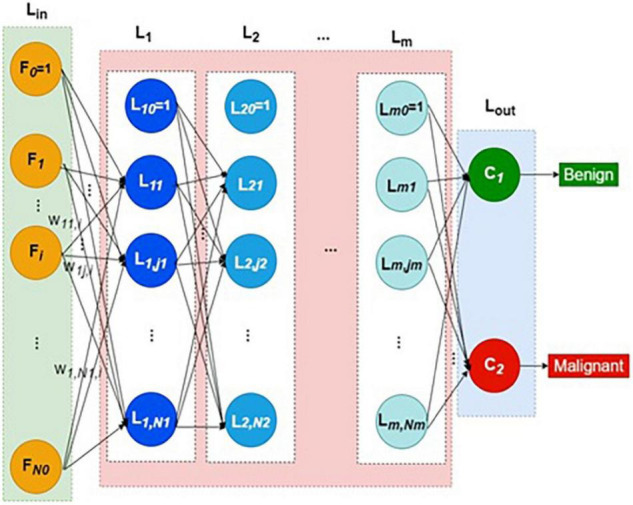
Structure of deep neural network.

Thus, each neuron except bias in the first hidden layer computes the weighted sum of all input layer signals. The weight vector reaching each hidden neuron is independent of all other weight vectors. Each layer *l*_*k*_ has *N*_*k*_ number of hidden neurons plus one bias neuron. The structure of DNN is shown in Figure 1. The collection of all weight vectors belonging to a particular layer index *k* is called weight matrix *W^k^*. Similarly, each hidden layer *L*_*k*_ is connected with its previous layer by its weight matrix *W^k^*.

Thus, every hidden layer indexed as *k* = *1* to *m* is connected by weight matrix *W^k^* from the layer *k*–1, as defined in Equations (1–4)


(1) $$n\,e\,t_{j,p}^{k}=w_{j,1}^{k}\,x_{1,p}+w_{j,2}^{k}\,x_{2,p}+\cdots +w_{j,N_{k}}^{k}\,x_{N_{k},p}+w_{j,0}^{k}$$

(2) $$n\,e\,t_{j,p}^{k}=\sum_{l=0}^{m}w_{j,l}^{k}\,x_{j,p}$$

$$y_{j,p}^{k}=n\,e\,t_{j,p}^{k},i\,f\,n\,e\,t_{j,p}^{k}>0$$

(3) =0,o⁢t⁢h⁢e⁢r⁢w⁢i⁢s⁢e⁢(R⁢E⁢L⁢U⁢f⁢u⁢n⁢c⁢t⁢i⁢o⁢n)

Generally, the weight matrix is defined as in Equation (4),


(4) $$W^{k}=\left(\begin{array}{ccc} w_{1,0}^{k} & w_{1,1}^{k} & \begin{array}{cc} \cdots & w_{1,N\,k-1}^{k} \end{array}\\ w_{2,0}^{k} & w_{2,1}^{k} & \begin{array}{cc} \cdots & w_{2,N\,k-1}^{k} \end{array}\\ \begin{array}{c} \vdots \\ w_{N\,k,0}^{k} \end{array} & \begin{array}{c} \vdots \\ w_{N\,k,1}^{k} \end{array} & \begin{array}{cc} \begin{array}{c} \vdots \\ \cdots \end{array} & \begin{array}{c} \vdots \\ w_{N\,k,N\,k-1}^{k} \end{array} \end{array} \end{array}\right)$$

where *k* is layer index, *j* refers to the *j*-th neuron in any layer *k*, *N*_*k*_ represents the number of hidden neurons in the *k*-th layer, *p* is the image or pattern in general, and *w^k^_j_*,_l_ refers to the weight connecting the *l*-th neuron of layer *k* − 1 to the *j*-th neuron of layer *k*.

The output of the last hidden layer feeds to the output layer, *L*_out_. The output layer has just two neurons representing benign and malignant classes. The softmax function is the activation function in the output layer, defined in Equations (5–7).


(5) $$z_{q}=\sum_{j=0}^{N\,m}w_{q,j}^{m}\,y_{j,p}^{m}$$

where *q* = 1 refers to malignant and *q* = 0 refers to benign classes.


(6) C=qsoftmax(z)q

(7) $${\text{Softmax}}_{z_{q}}=\frac{e^{z_{q}}}{\sum_{r=0}^{1}e^{z_{r}}}$$

The cross-entropy loss (CEL) cost function is defined as in Equation (8).


(8) $$\text{CEL}=-\sum_{q=0}^{1}t_{i}\,\text{log}\,\left(C_{q}\right)$$

where *t*_*i*_ refers to target value, which can take the value of (0,1).

For the classification process, this study uses the classical ANN because a lot of preprocessing has been performed through various units like feature extraction and selections, segmentation using a DNN. Hence, the proposed classifier acts as a combiner of different classifiers.

In any case, as the classifier evaluates the relative effectiveness of the architectures, it is employed to combine the proposed outputs of various units using the classical multilayer perceptron (MLP).

The activation function for all the hidden layers is the Rectified linear unit (RELU) function. The activation function for the output layer is the softmax function. The cost function is the CEL function. In this configuration, the classifiers are trained by applying the traditional backpropagation algorithm.

### Least Square Fit

In the proposed scheme, seven different classifiers use the same input and produce their outputs. These outputs are the same most of the time and differ in some regions of the data space. A common judgment can be arrived at by the weighted sum of all these outputs. The vector of these weights (along with the bias) that minimizes sum squared error (SSE) can be obtained using the Least Square Fit (LSF) method.

### The Phases of Classifier Development

**FIGURE 2:**
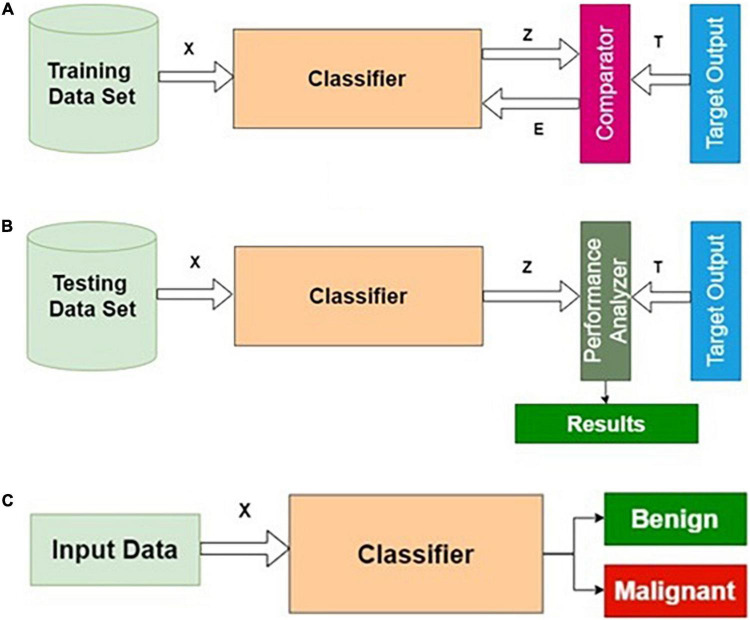
Phases of classifier development: **(A)** training, **(B)** testing, and **(C)** operational phases.

Training, testing, and normal operation are the three important phases for any classifier in general. The training and testing processes are repeatedly carried out until the results of the training performance are satisfactory. The phases of classifier development, the essence of this procedure, are shown in Figure 2.

#### Training Algorithm

**Step 1**: Initialization: All weights and biases corresponding to all layers are initialized to small random numbers [say, (− 0.3, + 0.3)].

**Step 2**: Forward computation.

**Step 3**: Adaptation of weights using the backpropagation algorithm.

**Step 4**: Termination using early stopping criteria.

#### Testing Algorithm

**Step 1**: Apply the testing dataset as input to the trained model.

**Step 2**: Compute the predicted output *Z*.

**Step 3**: Calculate the accuracy, specificity, and sensitivity using *T* and *Z*.

#### Operation Algorithm

**Step 1**: Apply the unknown query image *I*_*r*_ as input to the validated model.

**Step 2**: Compute *Z*_*r*_ for the query image *I*_*r*_.

**Step 3**: Return *Z*_*r*_ as the predicted output.

### Proposed Architectures for Brain Tumor Classification

For image classification, two possible approaches exist in current practice, namely, image-based and feature-based classifications.

In image-based classification, the input is the actual image. The features required for accurate classification are derived using the many-layered module, which entirely consists of the hidden layers. The final feature vectors from this module are classified in the output layer. The output layer combines these feature vectors with the classifier outputs.

In the next approach, namely, feature-based classification, vectors of features derived from each image are submitted to the classifier. These individual features are extracted mathematically, geometrically, and statistically from the properties of the image. These properties depend on the following dimensions: (i) color, (ii) pixel intensity, and (iii) pixel location. As a function of these dimensions, many features can be extracted. Out of these features, only a part of them is relevant for a particular problem. Hence, the feature selection process is used to select the minimal set of relevant and sufficient features for the existing problems.

The DL-based architectures that use (i) actual images or (ii) feature vectors derived from the actual images or (iii) a combination of both (i) and (ii) have been analyzed in this study for classifying the MRI brain tumor images into benign or malignant. The various proposed architectures for image classification are listed as follows:

Part I: Feature-based classification (FC)

i. Actual image feature-based classifier (AIFC)
ii. Segmented image feature-based classifier (SIFC)
iii. Actual and segmented image feature-based classifier (ASIFC).

Part II: Image-based classification (IC)

i. Actual image-based classifier (AIC)
ii. Segmented image-based classifier (SIC)
iii. Actual and segmented image-based classifier (ASIC).

Part III: Combined feature and image-based classification (CFIC)

This classification process works entirely based on feature extraction, selection, and classification. The Kaggle dataset is divided into training and testing datasets using random division with the different ratios given in Table 1. The feature vector is extracted from each training and testing dataset image. The model is trained using the training dataset. Approximately 50% of the testing dataset is used for validation. After satisfactory validation and early stopping, the testing is conducted using the testing dataset.

**TABLE 1:** Performance of different training/testing ratios.

Training (Tr)/testing (Te) ratio (%)	Accuracy (%)
90–10	95.28
80–20	92.87
70–30	98.97
60–40	95.52
50–50	94.91
40–60	92.46
30–70	90.22

**FIGURE 3:**
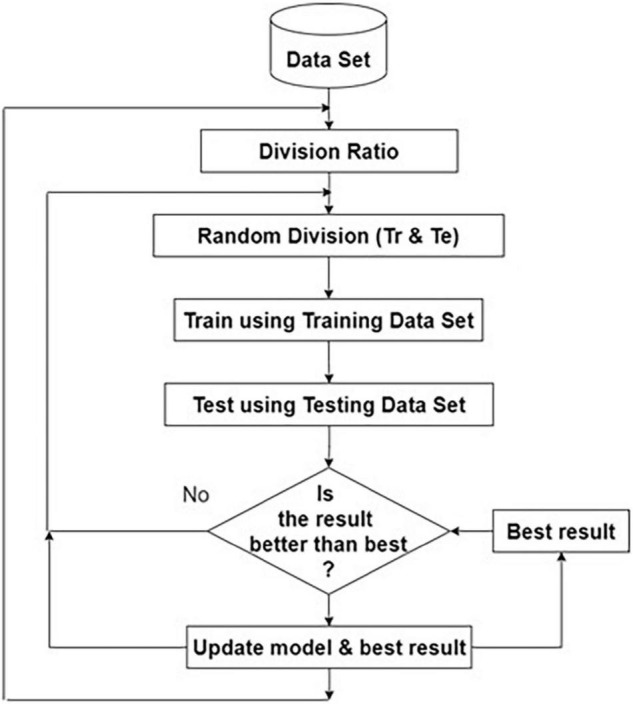
Flow diagram for choosing the best model.

The model parameters are stored and the same process is repeated for the same ratio for storing the best model. The same iterative procedure is repeated for different ratios of training, and testing dataset sizes. Finally, the best model from the stored models is chosen, as shown in Figure 3.

#### Feature-Based Classification

Deep neural networks are known for learning deep features, which are, in turn, derived from lower-order features. In this study, two possible scenarios exist, namely, AIFC and SIFC. Both approaches have their respective strength and limitations. This study considers the combinations of these two approaches and compares their relative performance. The stopping criteria of all the methods are the best combination of the training and testing images chosen from the dataset, and they are stored.

##### Actual Image Feature-Based Classifier

The preprocessed MRI brain original image features are used as input to the AIFC. The target data for the input image are labeled as benign or malignant. The AIFC classifier is trained using actual training image features and corresponding target outputs. A detailed description of the DNN has been presented in the “Deep Neural Network Architecture” section and the “The Phases of Classifier Development” section.

**FIGURE 4:**
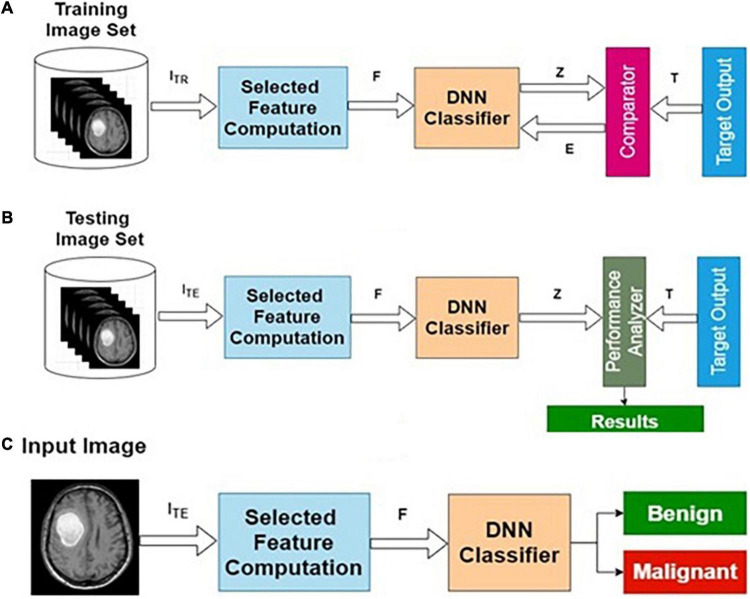
Actual image feature-based classifier (AIFC): **(A)** training, **(B)** testing, and **(C)** operational phases.

Table 2 presents the performance of AIFC with different training and testing ratios of MRI brain images. The AIFC architecture is shown in Figure 4. The best performance of the AIFC was observed for the ratio of 70:30 with scores of 96.10, 85.28, and 94.52% for sensitivity, specificity, and accuracy, respectively.

**TABLE 2:** Performance of AIFC with different training/testing ratios.

Training–testing ratio (%)	Sensitivity (%)	Specificity (%)	Accuracy (%)
90–10	91.33	89.01	90.43
80–20	88.36	96.79	91.04
70–30	96.10	85.28	94.52
60–40	93.59	85.15	91.85
50–50	92.33	84.96	90.63
40–60	89.07	77.59	86.35
30–70	88.97	72.18	84.11

##### Segmented Image Feature-Based Classifier

**FIGURE 5:**
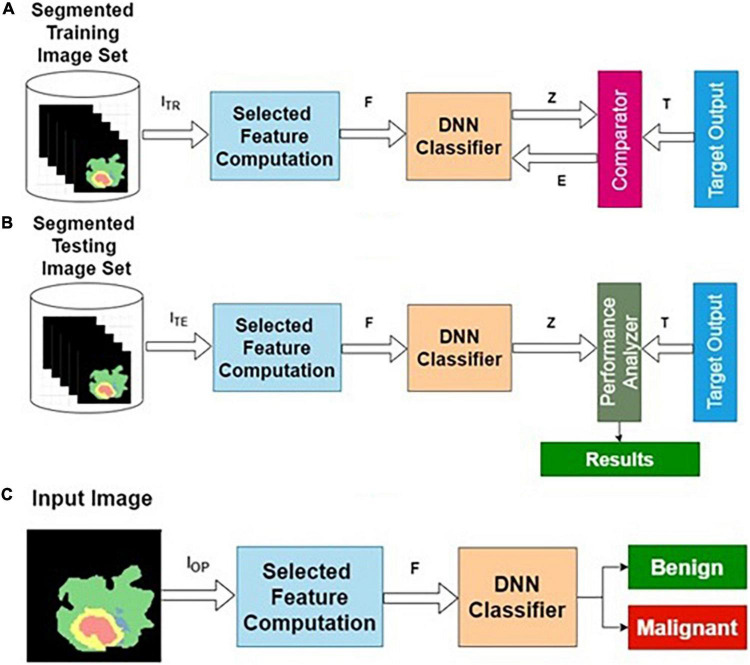
Segmented image feature-based classifier (SIFC): **(A)** training, **(B)** testing, and **(C)** operational phases.

The preprocessed MRI brain segmented image features are used to the DNN. The target data for the input images are labeled as benign or malignant. The DNN classifier is trained using segmented training image features and corresponding target outputs. The training, validation and testing are conducted using the procedure described in the “Deep Neural Network Architecture” section and the “The Phases of Classifier Development” section. The architecture for SIFC is presented in Figure 5.

Table 3 presents the performance of SIFC with different training and testing ratios of MRI brain images. The SIFC architecture is shown in Figure 5. The best performance of the SIFC is observed for the ratio of 70:30 with scores of 85.07, 78.31, and 87.06% for sensitivity, specificity and accuracy, respectively.

**TABLE 3:** Performance of SIFC with different training/testing ratios.

Training–testing ratio (%)	Sensitivity (%)	Specificity (%)	Accuracy (%)
90–10	84.67	86.82	87.17
80–20	83.27	75.31	86.19
70–30	85.07	78.31	87.06
60–40	84.49	77.31	85.34
50–50	82.17	72.70	82.69
40–60	80.93	70.22	80.04
30–70	80.67	69.08	79.43

##### Actual and Segmented Image Feature-Based Classifier

**FIGURE 6:**
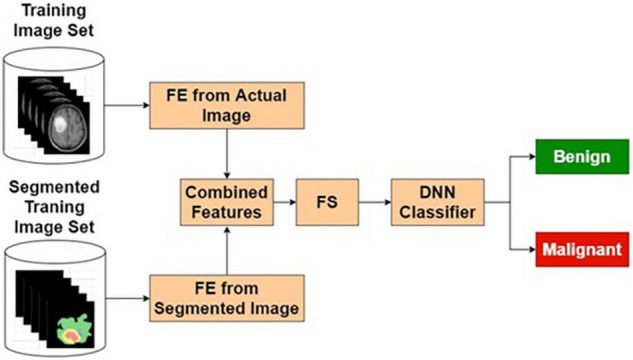
Actual and segmented image feature-based classifier.

The features extracted from the original image, being supervised in nature, may contain many relevant and irrelevant features. A classifier that exclusively uses the original image may have limited resolving power. In this study, it has been observed that classification performance based on the purely segmented image has some limitations. As a contrasting space compared with the original image, the segmented images are bound to produce features that ignore the common normal background aspects. Hence, combining these two classifiers is expected to produce better results. Based on these reasons, various combinational architectures are proposed. In this method, the classifier output from the original image-based classifier and segmented image-based classifier are combined to form a weighted output that is classified using an LSF technique shown in Figure 6.

Table 4 presents the performance of ASIFC with different training and testing ratios of MRI brain images. The ASIFC architecture is shown in Figure 6. The best performance of the ASIFC is observed for the ratio of 70:30 with scores of 97.57, 89.1, and 96.33% for sensitivity, specificity, and accuracy, respectively.

**TABLE 4:** Performance of ASIFC with different training/testing ratios.

Training–testing ratio (%)	Sensitivity (%)	Specificity (%)	Accuracy (%)
90–10	95.67	87.10	95.06
80–20	92.25	82.72	92.26
70–30	97.57	89.10	96.33
60–40	94.10	87.13	92.67
50–50	92.86	86.73	91.45
40–60	90.00	80.60	87.78
30–70	89.26	72.89	84.52

*ASIFC, actual and segmented image feature-based classifier.*

#### Image-Based Classification

Deep convolutional neural networks are well-known for their image processing capabilities. In this study, two possible scenarios exist, namely, AIC and segmented image-based classifier. Both approaches have their respective strengths and limitations. In this study, the combinations of these two approaches are investigated, and their performances are compared.

##### Actual Image-Based Classifier

**FIGURE 7:**
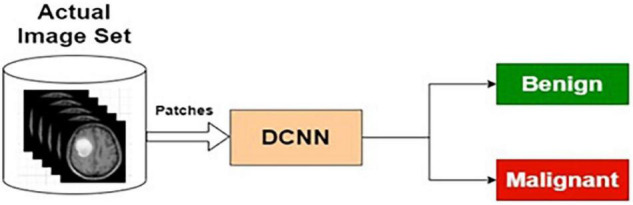
Actual image-based classifier.

The preprocessed MRI brain original images are used as input to the AIC. The target data for the input images are labeled as benign or malignant. The AIC classifier is trained using actual training images and corresponding target outputs. The training, validation, and testing are conducted using the procedure described in the “Deep Neural Network Architecture” section and the “The Phases of Classifier Development” section. The architecture for AIC is presented in Figure 7.

Table 5 presents the performance of AIC with different training and testing ratios of MRI brain images. The AIC architecture is shown in Figure 7. The best performance of the AIC is observed for the ratio of 70:30 with scores of 96.35, 85.92, and 95.85% for sensitivity, specificity, and accuracy, respectively.

**TABLE 5:** Performance of AIC with different training/testing ratios.

Training–testing ration (%)	Sensitivity (%)	Specificity (%)	Accuracy (%)
90–10	92.83	81.36	93.26
80–20	89.40	84.04	92.46
70–30	96.35	85.92	95.85
60–40	94.36	84.12	93.08
50–50	93.25	83.05	92.06
40–60	90.53	82.33	88.59
30–70	89.83	74.30	85.34

##### Segmented Image-Based Classifier

The preprocessed MRI brain segmented images are applied as input to the DCNN. The target data for the input images are labeled as benign or malignant. The DCNN classifier is trained using segmented training images and corresponding target outputs.

**FIGURE 8:**
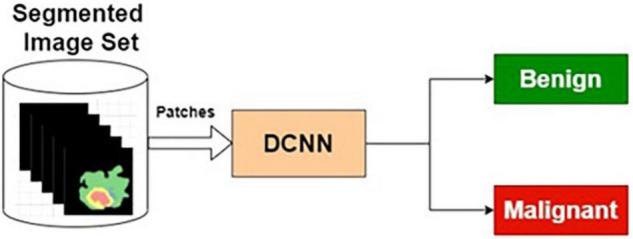
Segmented image-based classifier.

Table 6 presents the performance of *SIC* with different training and testing ratios of MRI brain images. The *SIC* architecture is shown in Figure 8. The best performance of the *SIC* is observed for the ratio of 70:30 with scores of 95.43, 84.42, and 92.67% for sensitivity, specificity, and accuracy, respectively.

**TABLE 6:** Performance of *SIC* with different training/testing ratios.

Training–testing ratio (%)	Sensitivity (%)	Specificity (%)	Accuracy (%)
90–10	92.83	83.08	89.37
80–20	88.42	82.63	88.39
70–30	95.43	84.42	92.67
60–40	91.03	82.25	90.78
50–50	89.55	80.66	89.35
40–60	86.40	78.97	87.28
30–70	90.26	75.35	85.95

##### Actual and Segmented Image-Based Classifier

The pre-processed MRI brain original and segmented images are used as input to the AIC and *SIC*, respectively. The target data for each classifier are labeled as benign or malignant, based on the input images. The AIC and *SIC* classifiers are combined using an LSF combiner.

**FIGURE 9:**
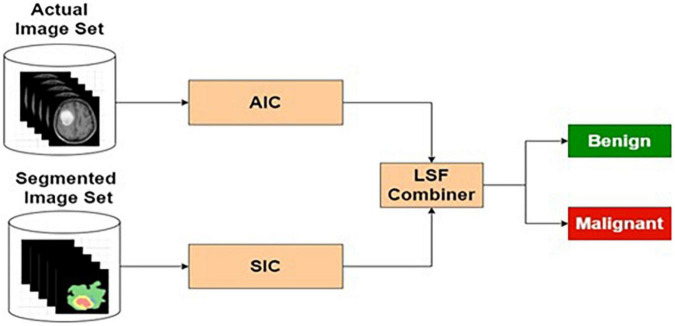
Actual and segmented image-based classifier.

Table 7 presents the performance of ASIC with different training and testing ratios of MRI brain images. The ASIC architecture is shown in Figure 9. The best performance of the ASIC is observed for the ratio of 70:30 with scores of 97.79, 95.32, and 97.56% for sensitivity, specificity, and accuracy, respectively.

**TABLE 7:** Performance of ASIC with different training/testing ratios.

Training–testing ratio (%)	Specificity (%)	Specificity (%)	Accuracy (%)
90–10	95.00	94.62	95.46
80–20	91.55	92.36	92.67
70–30	97.79	95.32	97.56
60–40	95.13	91.09	94.30
50–50	94.58	92.48	94.09
40–60	91.73	86.21	90.43
30–70	93.12	82.39	90.02

#### Combined Feature and Image-Based Classification

**FIGURE 10:**
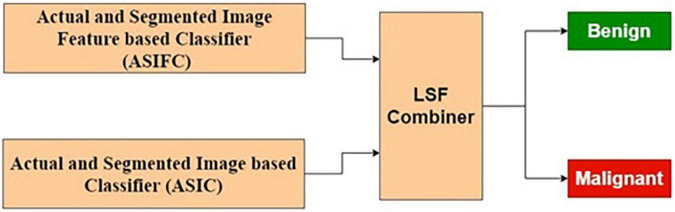
Combined feature and image-based classifier.

The ASIFC explained in the “Actual and Segmented Image Feature-Based Classifier” of the section “Feature-Based Classification” and the ASIC explained in the “Feature-Based Classification” of the section “Image-based classification” are combined using LSF combiner. The LSF combiner produces the predicted output as a weighted sum of ASIFC, ASIC, and a bias. These weights are learned from the target output and the input image to minimize the squared error. The architecture is shown in Figure 10.

Table 8 presents the performance of CFIC with different training and testing ratios of MRI brain images. The CFIC architecture is shown in Figure 10. The best performance of the CFIC is observed for the ratio of 70:30 with scores of 98.86, 97.14, and 98.97% for sensitivity, specificity, and accuracy, respectively.

**TABLE 8:** Performance of CFIC with different training/testing ratios.

Training–testing ratio (%)	Sensitivity (%)	Specificity (%)	Accuracy (%)
90–10	96.67	96.67	95.28
80–20	93.70	95.68	92.87
70–30	98.86	97.14	98.97
60–40	95.90	94.06	95.52
50–50	95.11	94.25	94.91
40–60	93.07	90.52	92.46
30–70	93.27	82.75	90.22

## Results and Discussion

The Kaggle Brain Tumor Detection 2020 dataset has been used to train and test the proposed classifiers. The proposed seven architectures for the classification of MRI brain tumor images are described in the “Proposed Architectures for Brain Tumor Classification” section. This section tabulates and discusses comparison of the performance of the proposed architectures.

The performance evaluation metrics are as follows:


(9) $$\text{Sensitivity}=\,\frac{T\,P\,p}{T\,P\,p+F\,N\,p}$$

(10) $$\text{Specificity}=\frac{T\,N\,p}{T\,N\,p+F\,P\,p}$$

(11) $$\text{Accuracy}=\,\frac{T\,P\,p+T\,N\,p}{T\,P\,p+F\,N\,p+T\,N\,p+F\,P\,p}$$

where TP_*p*_ is the malignant image correctly predicted as malignant, TN_*p*_ is the benign image correctly predicted as benign, FP_*p*_ is the benign image incorrectly predicted as malignant, and FN_*p*_ is the malignant image incorrectly predicted as benign.

### Comparison of the Performance of Proposed and Investigated Classification Methods

**FIGURE 11:**
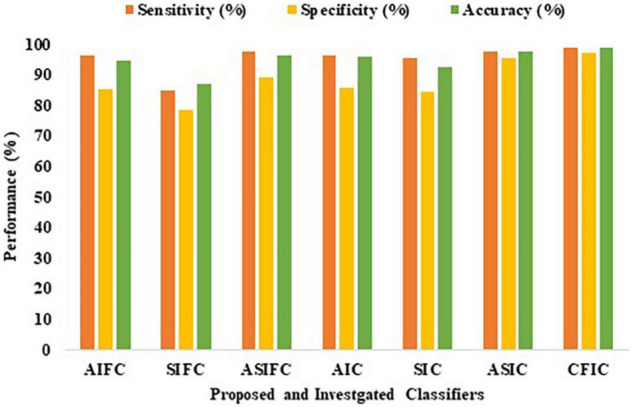
Performance of proposed and investigated classifiers.

Table 9 shows the comparison of the performance of the various proposed methods based on sensitivity, specificity, and accuracy. Out of all the proposed learning architectures, the CFIC outperforms all other methods. The proposed CFIC method gives significantly better sensitivity, specificity, and accuracy with scores of 98.86, 97.14, and 98.97%, respectively. Figure 11 presents a visual comparison of various classification methods.

**TABLE 9:** Comparison of the performance of proposed and investigated classification methods.

Proposed methods	Sensitivity (%)	Specificity (%)	Accuracy (%)
AIFC	96.10	85.28	94.52
SIFC	85.07	78.31	87.06
ASIFC	97.57	89.10	96.33
AIC	96.35	85.92	95.85
*SIC*	95.43	84.42	92.67
ASIC	97.79	95.32	97.56
CFIC	98.86	97.14	98.97

### Comparison of the Performance of Proposed and Existing Classification Methods

The performance of the proposed method is compared and tabulated using the various existing methods for brain tumor classification.

#### Comparison of Performance Using Sensitivity

**FIGURE 12:**
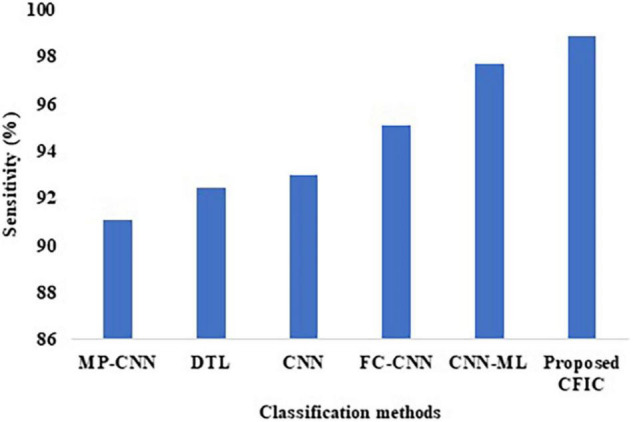
Sensitivity of classification methods.

The sensitivity defined in Equation (9) is used for the comparison of the performance. Table 10 shows that the sensitivity of the proposed CFIC technique is 98.86%, whereas, for the next best method, namely CNN-ML, the score is 97.72%. Thus, the proposed method shows an improvement of 1.14% over its nearest competitor. Figure 12 presents a visual comparison with the sensitivity of various classification methods.

**TABLE 10:** Comparison of the performance of the proposed and existing classification methods.

Methods	Sensitivity (%)	Specificity (%)	Accuracy (%)
MP-CNN	91.10	95.30	97.30
DTL	92.45	96.99	98.00
CNN	93.00	95.12	94.39
FC-CNN	95.09	93.67	91.43
CNN-ML	97.72	96.45	98.83
Proposed CFIC	98.86	97.14	98.97

#### Comparison of Performance Using Specificity

**FIGURE 13:**
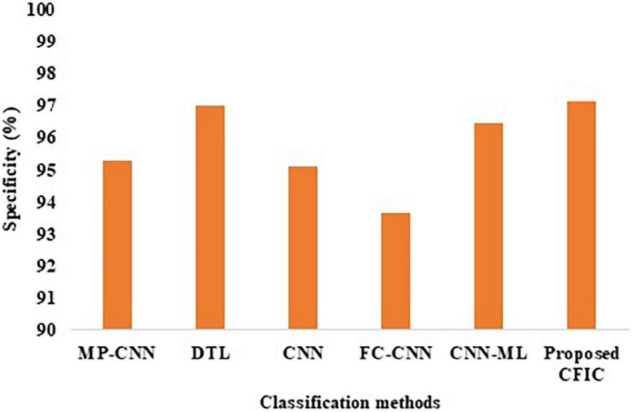
Specificity of classification methods.

The specificity defined in Equation (10) is used for the comparison of the performance. Table 10 shows that the specificity of the proposed CFIC method is 97.14%, whereas, for the next best method, namely DTL, the score is 96.99%. Thus, the proposed method shows an improvement of 0.15% over its nearest competitor. Figure 13 presents a visual comparison with the specificity of various classification methods.

#### Comparison of Performance Using Accuracy

**FIGURE 14:**
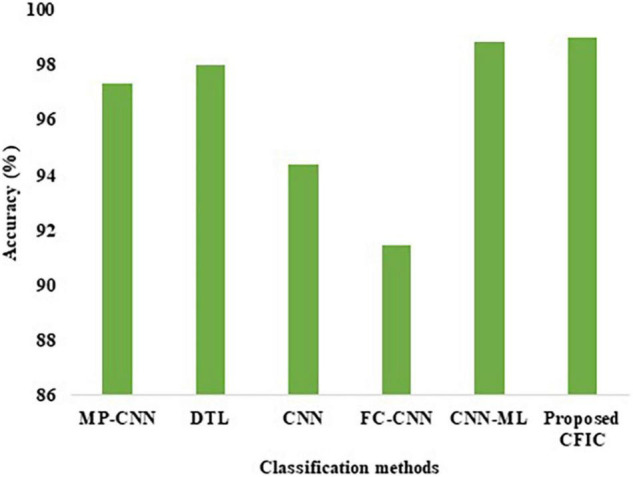
Accuracy of the classification methods.

The accuracy defined in Equation (11) is used for the comparison of the performance. Table 10 shows that the accuracy of the proposed CFIC scheme is 98.97%, whereas, for the next best method, namely CNN-ML, the score is 98.83%. Thus, the proposed method shows an improvement of 0.14% over its nearest competitor. Figure 14 presents a visual comparison with the accuracy of various classification methods.

The classifier, namely CFIC, is better in performance than AIFC, SFIC, ASIFC, AIC, *SIC*, and ASIC classifiers using the Kaggle Brain Tumor Detection 2020 dataset in terms of accuracy.

## Conclusion and Future Work

For the classification problem, seven different combinational architectures are investigated, each showing improvements in terms of one or more performance metrics. The AIFC is performing better than the SIFC. The ASIFC is performing better than both AIFC and SIFC. The implication is that the border information is last in a segmented image. The AIC is performing better than the *SIC*. The ASIC is better than both AIC and *SIC*. However, the CFIC is performing better when combined with ASIFC and ASIC in the accuracy. Finally, the CFIC method is the best among the proposed classification methods. The proposed DNN-based CFIC method gives significantly better results in terms of sensitivity, specificity, and accuracy with 98.86, 97.14, and 98.97%, respectively, compared with the next best existing techniques, namely CNN-ML and DTL with 97.72, 96.99, and 98.83%, respectively. The following are the directions for future work: (a) The algorithms developed should be incorporated into the software used by physicians and (b) the methods and techniques propounded in this study can only be applied to gray images. Further work could employ color images for the same problems.

## Data Availability Statement

The original contributions presented in the study are included in the article/supplementary material, further inquiries can be directed to the corresponding authors.

## Author Contributions

AV, SM, and GM contributed in problem formulation, implementation and results. KK, JS, VV, and VS contributed in methodology identification and techniques to project results. All authors contributed to the article and approved the submitted version.

## Conflict of Interest

The authors declare that the research was conducted in the absence of any commercial or financial relationships that could be construed as a potential conflict of interest.

## Publisher’s Note

All claims expressed in this article are solely those of the authors and do not necessarily represent those of their affiliated organizations, or those of the publisher, the editors and the reviewers. Any product that may be evaluated in this article, or claim that may be made by its manufacturer, is not guaranteed or endorsed by the publisher.
